# Editorial: Biology-Driven Targeted Therapy of Pediatric Soft-Tissue and Bone Tumors: Current Opportunities and Future Challenges

**DOI:** 10.3389/fonc.2016.00039

**Published:** 2016-02-18

**Authors:** Thomas G. P. Grünewald, Simone Fulda

**Affiliations:** ^1^Laboratory for Pediatric Sarcoma Biology, Institute of Pathology, Ludwig Maximilian University of Munich, Munich, Germany; ^2^Institute for Experimental Cancer Research in Pediatrics, Goethe-University Frankfurt, Frankfurt, Germany; ^3^German Cancer Consortium (DKTK), Heidelberg, Germany; ^4^German Cancer Research Center (DKFZ), Heidelberg, Germany

**Keywords:** osteosarcoma, Ewing sarcoma, rhabdomyosarcoma, epithelioid sarcoma, targeted therapy, immunotherapy, cell death, preclinical models

**The Editorial on the Research Topic**

**Biology-Driven Targeted Therapy of Pediatric Soft-Tissue and Bone Tumors: Current Opportunities and Future Challenges**

Sarcomas constitute a large and diverse group of malignant neoplasias and the predominant group of non-CNS-related solid tumors in childhood and adolescence (1). Recent advances in the understanding of the genetic and biological basis of pediatric soft tissue and bone tumors, especially owing to the advent of “omics” technologies, have led to an exponential increase in the current knowledge on the genetic and cellular pathomechanisms that drive these diseases (2).

This offers the unprecedented opportunity to develop and implement targeted therapies, such as monoclonal antibodies, small molecules, and immunotherapies in standard and/or personalized treatment regimens (2). However, to date, only a few examples document a successful translation of discoveries from the bench to the bedside, which significantly improved patient outcome while having little adverse effects (3, 4). Recent reviews (5) and international expert congresses further emphasize the urgent need for a more rapid and especially more successful translational process (6–8) (Kovar et al., Schäfer et al.).

This *Frontiers Research Topic* entitled “*Biology-driven targeted therapy of pediatric soft-tissue and bone tumors: current opportunities and future challenges*” was dedicated to this aspect and provided a transdisciplinary forum for researchers working at the interfaces between basic cell biology, tumorigenesis, and personalized medicine. Many excellent researchers have contributed to this topic now covering the most common but also rare sarcoma entities of this age group, such as osteosarcoma, Ewing sarcoma, rhabdomyosarcoma, and epithelioid sarcoma.

In order to accelerate the development of novel targeted therapeutics, suitable genetically engineered animal models and xenograft models are required. In this regard, Geier et al. discuss preclinical human tumor xenograft models of pediatric sarcomas that may be used practically to identify novel agents and how “omics” approaches may be implemented for identification of novel biomarkers, which can discriminate sensitive and resistant tumors to these agents. Since the ultimate goal of anticancer therapy is to kill cancer cells, it is important to assess in detail the modes of cell death in preclinical models. Rello-Varona et al. survey different modes of cell death and propose standards of how to adequately assess them, which is especially important in such a heterogeneous group of tumor entities such as human sarcomas.

Osteosarcoma is the most common pediatric bone cancer (1). Sampson et al. discuss current knowledge on the role of micro RNAs (miRNAs) and their target genes in osteosarcoma and evaluate their potential use as therapeutic agents. They also summarize the efficacy of inhibition of oncogenic miRNAs or expression of tumor suppressor miRNAs in preclinical models of osteosarcoma (Sampson et al.).

Ewing sarcoma is the second most common pediatric bone-associated sarcoma (1). Since its discovery in 1921 by James Ewing, the precise histogenesis of Ewing sarcoma remains enigmatic (9). Despite this histogenetic uncertainty, Ewing sarcoma is genetically well characterized by the presence of pathognomonic *EWSR1-ETS* gene fusions (usually *EWSR1-FLI1*) (10), which drive this disease by acting as oncogenic transcription factors (10). In their review, Cidre-Aranaz and Alonso assess five major EWSR1-FLI1 target genes, their signaling pathways, and shed light on how these pathways could be exploited therapeutically. Indeed, earlier work showed that some EWSR1-FLI1 target genes are very specifically expressed in Ewing sarcoma relative to normal tissues (11), thus possibly constituting attractive targets for (immuno)-therapeutic intervention. In accordance, subsequent experiments showed that IL2 transgenic Ewing sarcoma cells elicit tumor-specific T and NK cell responses *in vitro* and *in vivo* (12). In a subsequent study, Reuter et al. now investigate the role of immunostimulation by OX40 ligand (also known as CD252 or tumor necrosis factor ligand family member 4) transgenic Ewing sarcoma cells. The authors found that OX40L expression in Ewing sarcoma cells enhanced immune stimulation, suggesting that the OX40/OX40L pathways should be considered in the design of immunotherapies against Ewing sarcoma (Reuter et al.). However, immunotherapeutic advances are not limited to Ewing sarcoma. Roberts et al. provide exiting new insights in immunotherapy of Ewing sarcoma and other pediatric sarcomas and point out the concept of integrating antibody-based and cell-based immunotherapy into an overall treatment strategy of sarcoma.

An innovative alternative approach for treatment of Ewing sarcoma is targeting the tumor’s micro-environment, instead of targeting the tumor cells directly. In fact, bone lesions from primary or metastatic Ewing sarcoma are characterized by extensive bone remodeling and osteolysis. Redini and Heymann expand on this important aspect and propose targeting the bone tumor micro-environment in Ewing sarcoma using osteoclast inhibitors, such as bisphosphonates and antagonists of receptor activator of NF-kappa B ligand (RANKL). In addition to this, Deel et al. summarize the known molecular alterations within the Hippo pathway in sarcomas and highlight how several pharmacologic compounds have shown activity in modulating Hippo components, providing proof-of-principle that Hippo signaling may be harnessed for therapeutic application in sarcomas.

Rhabdomyosarcomas are the most common soft tissue tumors of childhood (1) and constitute a heterogeneous group of cancers with myogenic differentiation featuring diverse cytogenetic and mutational aberrations (7). Hettmer et al. describe two disease-relevant mouse myogenic tumor models driven either by oncogenic *Kras* in *p16p19^*null*^* or by a mutant *Smoothened* allele. In line with this, Ridzewski et al. explore the therapeutic value of four *Smoothened* inhibitors in four different rhabdomyosarcoma cell lines. They found that some inhibitors induced strong proapoptotic and antiproliferative effects in some rhabdomyosarcoma cell lines, while others paradoxically induced cellular proliferation at certain concentrations (Ridzewski et al.). Because of this heterogeneous response, the authors propose to conduct pretesting of *Smoothened* inhibitors in patient-derived short-term rhabdomyosarcoma cultures or patient-derived xenograft mouse models before applying these drugs to patients (Ridzewski et al.). In an accompanying article, Schott et al. provide evidence that oncogenic *RAS* mutants confer resistance of RMS13 rhabdomyosarcoma cells to oxidative stress-induced ferroptotic cell death, which has important implications for the development of targeted therapies for rhabdomyosarcoma and which might at least partially explain heterogeneous responses on drug treatment depending on the *RAS* mutation status.

Noujaim et al. summarize clinically relevant biomarkers (e.g., SMARCB1, CA125, dysadherin, and others) with respect to targeted therapeutic opportunities for epithelioid sarcoma, which is a soft tissue sarcoma of children and young adults for which the preferred treatment for localized disease is wide surgical resection. However, current treatment regimens for epithelioid sarcoma still lack systematic coherence, and medical management is to a great extent undefined, which is why especially for patients with regional and distal metastases, the development of targeted therapies is greatly desired (Noujaim et al.). Noujaim et al. also examine the role of EGFR, mTOR, and polykinase inhibitors (e.g., sunitinib) in the management of local and disseminated disease. The authors propose to build a consortium of pharmaceutical, academic, and non-profit collaborators so that a roadmap can be developed toward effective biology-driven therapies of epithelioid sarcoma (Noujaim et al.). We believe that this *Frontiers Research Topic* has provided an excellent platform on which such consortia can be built on.

We anticipate that the data presented in the aforementioned original and review articles will be of great value for the scientific community to ultimately improve patient care and outcome. The success of this *Frontiers Research Topic* would not have been possible without the outstanding contribution of excellent scientists that served either as peer reviewers or additional guest associate editors.

## Author Contributions

All authors listed, have made substantial, direct and intellectual contribution to the work, and approved it for publication.

## Conflict of Interest Statement

The authors declare that the research was conducted in the absence of any commercial or financial relationships that could be construed as a potential conflict of interest.
